# Access to dental care for children with special healthcare needs: general dentists’ perspectives from the UAE

**DOI:** 10.3389/froh.2026.1928168

**Published:** 2026-08-06

**Authors:** Sara Mustafa, Anas AlSalami, Mawlood Kowash, Iyad Hussein, Amar Omer, Manal Al Halabi

**Affiliations:** Hamdan Bin Mohammed College of Dental Medicine, Mohammed Bin Rashid University of Medicine and Health Sciences (MBRU), Dubai Health, Dubai, United Arab Emirates

**Keywords:** access to care, general dentists, health disparities, special care dentistry, special healthcare needs, UAE

## Abstract

**Background:**

Children with special healthcare needs (SHCN) experience persistent disparities in access to oral healthcare. Despite legislative frameworks in the United Arab Emirates (UAE) supporting equitable care, provider-level barriers remain insufficiently characterized.

**Objective:**

To evaluate general dental practitioners' (GDPs) perceptions of barriers to delivering dental care to children with SHCN in the UAE and to examine associated demographic factors.

**Methods:**

A cross-sectional online survey was conducted among GDPs licensed across the UAE health authorities using a structured questionnaire adapted from a previously validated instrument. Data were collected between January and July 2025 through professional email lists, social media, conferences, and professional networks. Because recruitment used open dissemination channels, the total number of dentists who received or viewed the invitation could not be determined, and a conventional response rate could not be calculated. Descriptive statistics and exploratory subgroup analyses were used to summarize responses and examine associations.

**Results:**

A total of 206 GDPs participated. The most commonly reported barriers were patient-related behavioral challenges (53.4%) and insufficient practitioner training (52.9%), followed by communication difficulties (45.1%). Although most respondents reported that their clinics were physically accessible (86.4%), a majority indicated inadequate equipment (58.3%). Only 43.2% were aware of national legislation supporting individuals with disabilities. GDPs reported greater confidence in providing preventive and routine care compared to more complex procedures.

**Conclusion:**

UAE GDPs reported multiple **barriers** related **to** education**, communication,** behavior**, and clinical readiness in** providing dental care for children with SHCN. Although many respondents supported additional training and reported physical clinic accessibility, self-reported confidence was generally moderate, and suitable equipment was not universally available. These findings suggest the need for strengthened foundational undergraduate exposure, targeted continuing professional development, improved referral pathways, and context-sensitive clinic-readiness initiatives.

## Introduction

An estimated 1.3 billion people—approximately 16% of the global population—live with disability, and the World Health Organization (WHO) continues to report substantial health inequities affecting this population (1). Children with special healthcare needs (SHCN), defined by the American Academy of Pediatric Dentistry as those with physical, developmental, mental, sensory, behavioral, cognitive, or emotional impairment or limiting conditions requiring medical management, healthcare intervention, and/or specialized services, represent a particularly vulnerable subgroup; this definition also includes developmental or acquired orofacial conditions (2, 3). Children with SHCN experience disproportionate unmet dental need and oral health risk, compounded by functional limitations, behavioral challenges, complex care requirements, and dependence on caregivers (4, 5).

Access to dental care for children with SHCN is shaped by interacting patient-, provider-, and system-level factors. For this study, Andersen's Behavioral Model and the patient-centered access framework were used to guide interpretation of how practitioner characteristics, enabling resources, communication, clinic readiness, and perceived need may influence access to care (6, 7). These elements align directly with the study aims of assessing GDPs' perceived barriers and examining whether perceptions varied by practitioner characteristics.

Previous studies indicate that general dental practitioners' willingness to treat children with SHCN is influenced by perceived preparedness, confidence, prior clinical exposure, and patient cooperation (8, 9). Communication challenges, availability of suitable equipment, and referral support have also been identified as important barriers to service provision (10, 11). International evidence further suggests that insufficient undergraduate and postgraduate training in special care dentistry can reduce dentists' confidence and willingness to manage children with SHCN, particularly when behavioral or medical complexity is present (12, 13).

This issue is especially relevant in the United Arab Emirates (UAE), where national policy seeks an inclusive, barrier-free society for People of Determination and commits to accessible, high-quality healthcare and rehabilitation services (14). However, evidence on how general dental practitioners perceive barriers to treating children with SHCN in this setting remains limited. This study, therefore, aimed to assess UAE general dental practitioners' perceptions of barriers to providing dental care for children with SHCN and to examine whether these perceptions varied by practitioner characteristics.

## Materials and methods

### Study design

This cross-sectional survey evaluated general dental practitioners' (GDPs') perceptions of barriers to providing dental care to children with special healthcare needs (SHCN) in the United Arab Emirates (UAE). The study was designed and reported in accordance with the Strengthening the Reporting of Observational Studies in Epidemiology (STROBE) recommendations for cross-sectional studies (15).

### Setting and participants

The target population comprised GDPs licensed to practice in the UAE by the relevant professional regulatory authorities, including the Dubai Health Authority (DHA), Health Authority Abu Dhabi/SEHA (HAAD-SEHA), and the Ministry of Health and Prevention (MOHAP). Eligible participants were practicing GDPs registered with these authorities at the time of the survey. Exclusion criteria were undergraduate dental students, dental interns, postgraduate dental students, specialist dentists, dentists practicing outside the UAE, retired dentists, allied dental professionals, and individuals who declined participation.

### Sampling and recruitment

A minimum sample size of 240 GDPs was estimated using Cochran's formula for simple random sampling. Recruitment was conducted over a predefined seven-month period from January to July 2025. Despite repeated dissemination through email, social media, national conferences, and professional databases, 206 eligible complete responses were available for final analysis. Recruitment was stopped at the end of the planned data-collection period rather than after achieving the originally calculated target.

Overall, 271 survey submissions were received. Of these, 65 submissions (24.0%) were excluded before analysis: 23 questionnaires (8.5%) were incomplete, and 42 responses (15.5%) were from dentists who held licenses other than a GDP license. The final analytic sample therefore included 206 eligible complete responses (76.0% of received submissions).

The final sample size therefore fell below the planned estimate. This shortfall may have reduced statistical precision and power, particularly for subgroup comparisons where some categories contained small cell counts. Consequently, subgroup findings were interpreted cautiously and considered exploratory, with emphasis placed on the magnitude and direction of observed associations rather than statistical significance alone.

Because the survey link was distributed through open professional channels and could be forwarded among potential participants, the total number of GDPs who received or viewed the invitation could not be determined. Therefore, a conventional response rate could not be calculated. This limitation is acknowledged because it restricts assessment of participation and nonresponse bias.

### Data collection instrument

Data were collected using an anonymous English-language online questionnaire developed in Microsoft Forms. The instrument was adapted, with permission, from the previously validated survey by Adyanthaya et al. (11). The adaptation process retained the core domains of the original instrument while modifying the wording and contextual examples to improve relevance to the dental practice environment in the UAE. UAE-specific terminology and regulatory context were reviewed, including references to UAE health authorities and the national terminology of “People of Determination.”

The questionnaire included two sections. The first section captured demographic and professional characteristics, including sex, age, years of experience, nationality, country of graduation, practice setting, and types of SHCN patients encountered. The second section assessed perceived barriers, self-reported preparedness, confidence in treating children with SHCN, confidence in managing related medical emergencies, clinic accessibility, availability of suitable equipment, awareness of legislation, and interest in further training. Items included categorical, closed-ended, Likert-type, and matrix-format questions.

The online questionnaire used branching logic to ensure that respondents were presented only with items relevant to their previous answers. For example, follow-up questions on the types and frequency of dental treatment provided to children with SHCN were displayed only to respondents who indicated prior experience treating this patient group. Respondents who did not report such experience were directed to subsequent sections on perceived barriers, preparedness, referral patterns, and training needs. Mandatory consent and eligibility confirmation were required before survey access. Incomplete questionnaires and responses from individuals who did not meet the eligibility criteria were excluded before analysis.

### Validity and reliability

The questionnaire was adapted from a previously validated instrument that had been developed and used in a comparable Gulf-region context with broadly similar cultural and healthcare characteristics. Only minor wording modifications were made to align the instrument with the UAE healthcare system and local terminology, without changing the content, constructs, or response format of the original questionnaire. These modifications included UAE-specific terminology and regulatory context, such as references to UAE health authorities and the national terminology of “People of Determination.”

Because these modifications were limited and did not alter the underlying constructs, formal cultural adaptation and construct validity testing were not repeated. Nevertheless, the adapted questionnaire was reviewed by experts in pediatric dentistry and dental public health to ensure clarity, relevance, and appropriateness for the UAE context, supporting its face and content validity. The questionnaire was also pretested with 10 GDPs who met the eligibility criteria but were not included in the final analysis; this pretesting focused on clarity, completion time, terminology, and technical functionality rather than formal psychometric validation.

Internal consistency reliability of relevant multi-item domains was assessed using Cronbach's alpha. Alpha coefficients were calculated separately for the perceived barriers, confidence/preparedness, and training-related domains where applicable. For each domain, the number of items, corrected item–total correlations, and Cronbach's alpha if an item was deleted were examined. The domain “Criteria to refer to specialist care” included six items. Corrected item–total correlations indicated acceptable item contribution, and Cronbach's alpha did not improve after deletion of any item; therefore, all six items were retained in the final analysis. The absence of repeated construct validity testing has been acknowledged as a limitation, and future studies may consider more extensive psychometric evaluation of the adapted questionnaire in the UAE context.

### Data collection procedure

The questionnaire was administered in English and required approximately 10 min to complete. Participation was voluntary and anonymous, and no directly identifying personal data were collected. Before accessing the questionnaire, participants viewed a study information page outlining the study purpose, eligibility criteria, expected completion time, confidentiality safeguards, and consent process. Electronic informed consent was obtained using a mandatory checkbox before survey access. To strengthen confidentiality, subgroup analyses were not reported for cells with fewer than 10 observations.

### Outcome measures and variables

The primary outcome was GDPs' perceived barriers to providing dental care for children with SHCN. Secondary measures included self-reported adequacy of undergraduate preparation, interest in additional training, confidence in treating children with SHCN, confidence in managing related medical emergencies, clinic accessibility, availability of suitable equipment, and awareness of UAE legislation relevant to people of determination. Explanatory variables included sex, age group, years of experience, nationality, country of graduation, emirate of practice, and practice setting.

### Bias and data quality

Several procedures were used to support data quality, including predefined eligibility criteria, anonymous self-administration, mandatory electronic consent, and the exclusion of incomplete or ineligible responses prior to analysis. However, the use of convenience sampling may have introduced selection bias. GDPs with greater interest in SHCN care, stronger views about access barriers, or greater engagement with professional networks may have been more likely to participate. Conversely, dentists with limited interest, time constraints, lower digital engagement, or who practice in less-represented settings may have been underrepresented.

Because the denominator of dentists who received or viewed the invitation could not be established, nonresponse bias could not be quantified. This limits the ability to assess whether respondents differed systematically from nonrespondents. These issues reduce external validity and mean that the findings should be interpreted as reflecting the perceptions of participating GDPs rather than those of all GDPs practicing in the UAE.

### Ethical considerations

The study was conducted in accordance with the Declaration of Helsinki, Good Clinical Practice, and applicable UAE regulations governing human-participant research. Ethical approval was obtained from the Institutional Review Board of Mohammed Bin Rashid University of Medicine and Health Sciences on 29 December 2021 (MBRU-IRB-2021-89). Electronic informed consent was obtained from all participants before survey access.

### Statistical analysis

Survey data were exported from Microsoft Forms to Microsoft Excel and analyzed using IBM SPSS Statistics, version 28. Categorical variables were summarized as frequencies and percentages. Continuous variables were summarized using means and standard deviations when approximately normally distributed. Ordinal and Likert-type variables were summarized using frequencies, percentages, and summary scores as appropriate.

Normality of continuous or summary-scale variables was assessed using the Kolmogorov–Smirnov test. Associations between categorical variables were examined using Chi-square tests or Fisher's exact tests, as appropriate. Mann–Whitney U tests were used for comparisons involving ordinal or non-normally distributed outcomes.

The study was designed as a descriptive cross-sectional survey rather than an explanatory or prediction study. Accordingly, the statistical analyses were selected to address the study objectives by comparing groups and identifying statistically significant associations between practitioner characteristics, perceived barriers, treatment patterns, and referral-related responses. Chi-square tests, Fisher's exact tests, and Mann–Whitney U tests were used as appropriate. Observed subgroup differences were interpreted as associations and not as causal effects or independent predictors.

Multivariable logistic or ordinal regression analyses were not performed because identifying independent predictors and controlling for potential confounding were beyond the scope of this descriptive study. Effect-size estimates were not calculated for the exploratory subgroup analyses and are therefore not reported. Because multiple subgroup comparisons were exploratory and hypothesis-generating rather than confirmatory, adjustment for multiple testing was not applied; this decision avoided increasing the risk of Type II error but may have increased the possibility of Type I error. Findings were therefore interpreted cautiously, with emphasis on the direction and consistency of associations rather than *p*-values alone. All statistical tests were two-sided, and *p* < 0.05 was considered statistically significant.

## Results

### Participant characteristics

A total of 206 GDPs were included in the analysis. Most respondents were female (72.8%), aged <40 years (80.5%), UAE graduates (68.4%), and had >5 years of clinical experience (61.9%). Nearly half practiced in the government sector (47.6%) (Table 1).

**Table 1:** Demographic and professional characteristics of participating general dental practitioners in the United Arab Emirates (*n* = 206).

Variable	*n* (%)
Sex	
Male	56 (27.2)
Female	150 (72.8)
Age group (years)	
<40	165 (80.5)
≥40	40 (19.5)
Years of experience	
≤5 years	77 (38.1)
>5 years	125 (61.9)
Country of graduation	
UAE	141 (68.4)
Non-UAE	65 (31.6)
Practice setting	
Government	98 (47.6)
Private	86 (41.7)

Data are presented as frequency (percentage). Denominators vary because of missing responses for some variables.

GDP, general dental practitioner; UAE, United Arab Emirates.

### Internal consistency of questionnaire domains

Internal consistency was acceptable to good across the questionnaire's multi-item domains. Cronbach's alpha coefficients ranged from 0.76 to 0.84. The perceived barriers domain demonstrated good internal consistency (*α* = 0.81), the confidence/preparedness domain demonstrated good reliability (*α* = 0.84), and the training-related domain showed acceptable reliability (*α* = 0.76).

### Exposure to children with SHCN

**Figure 1:**
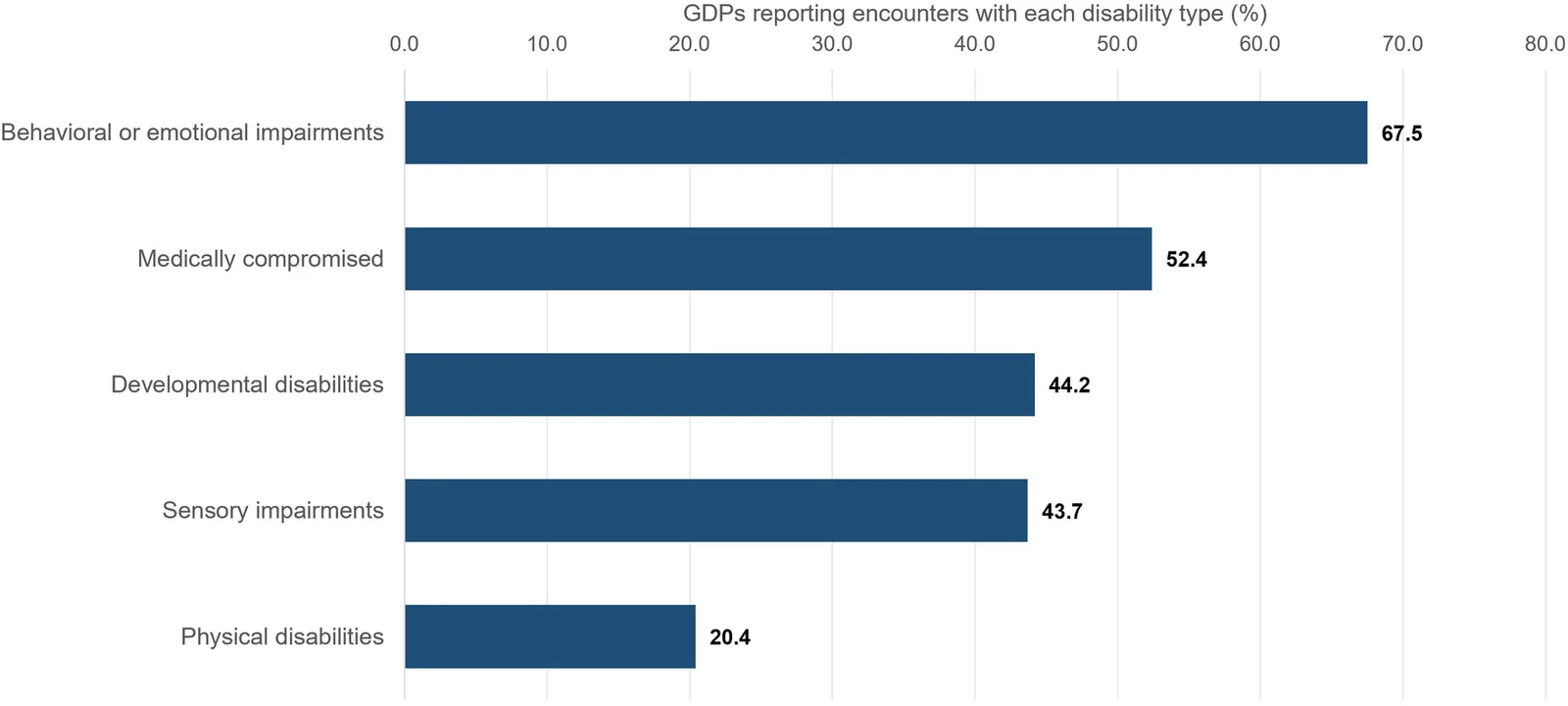
Types of disabilities among patients with special healthcare needs encountered by general dental practitioners in the United Arab Emirates.

The most frequently encountered special healthcare needs (SHCN) presentations were behavioral or emotional impairments (67.5%), followed by medically compromised conditions (52.4%), developmental disabilities (44.2%), sensory impairments (43.7%), and physical disabilities (20.4%) (Figure 1). Most GDPs (62.0%) reported seeing fewer than three children with SHCN per month.

### General perceptions and preparedness

Perceptions regarding training and preparedness are summarized in Table 2. Undergraduate preparation was rated as fair (41.3%). Most participants supported the inclusion of special care dentistry in undergraduate curricula (78.6%) and expressed interest in additional training (83.5%). Self-reported confidence was predominantly moderate for both treating children with SHCN (32.0%) and managing related medical emergencies (31.6%).

**Table 2:** GDP-reported training, confidence, and clinic readiness for managing children with special healthcare needs (SHCN) (*n* = 206).

Variable	*n* (%)
Dental education	
Adequacy of undergraduate dental education for managing patients with SHCN	
Poor	24 (11.7)
Fair	85 (41.3)
Good	65 (31.6)
Excellent	32 (15.5)
Mean ± SD	2.52 ± 0.89
Category	Fair to good
SCD should be part of the undergraduate curriculum	
No	6 (2.9)
Yes, theoretical	14 (6.8)
Yes, clinical	24 (11.7)
Yes, both	162 (78.6)
Interest in further training	
No	34 (16.5)
Yes	172 (83.5)
Dentist-related factors	
Confidence in treating children with SHCN	
Not confident	21 (10.2)
Slightly confident	57 (27.7)
Moderately confident	66 (32.0)
Confident	48 (23.3)
Highly confident	14 (6.8)
Mean ± SD	2.89 ± 1.09
Category	Slightly to moderately confident
Supporting staff comfort treating SHCN patients in the clinic	
Not comfortable	14 (6.8)
Slightly comfortable	42 (20.4)
Moderately comfortable	57 (27.7)
Comfortable	76 (36.9)
Highly comfortable	17 (8.3)
Mean ± SD	3.19 ± 1.07
Category	Moderately comfortable
Confidence in managing medical emergencies for SHCN children	
Not confident	37 (18.0)
Slightly confident	57 (27.7)
Moderately confident	65 (31.6)
Confident	38 (18.4)
Highly confident	9 (4.4)
Mean ± SD	2.64 ± 1.11
Category	Slightly to moderately confident
Health facility factors	
Clinic accessibility to SHCN patients	
No	28 (13.6)
Yes	178 (86.4)
Clinic with suitable equipment for SHCN patients	
No	120 (58.3)
Yes	86 (41.7)
Need to reduce disparities between SHCN patients and other children	
Strongly disagree	8 (3.9)
Disagree	17 (8.3)
Neutral	69 (33.5)
Agree	79 (38.3)
Strongly agree	33 (16.0)
Mean ± SD	3.54 ± 0.99
Category	Neutral to agree

Data are presented as frequency (percentage). Likert scale responses ranged from 1 (lowest) to 5 (highest).

GDP, general dental practitioner; SCD, special care dentistry; SHCN, special healthcare needs; SD, standard deviation.

Although 86.4% of GDPs reported that their clinics were accessible to children with SHCN, 58.3% indicated that their clinics lacked appropriate equipment. Equipment availability was not stratified by facility type in the present analysis. Awareness of UAE Federal Law No. 29 of 2006 (14) concerning the rights of people of determination was reported by 43.2% of respondents.

### Perceived barriers to care

**Figure 2:**
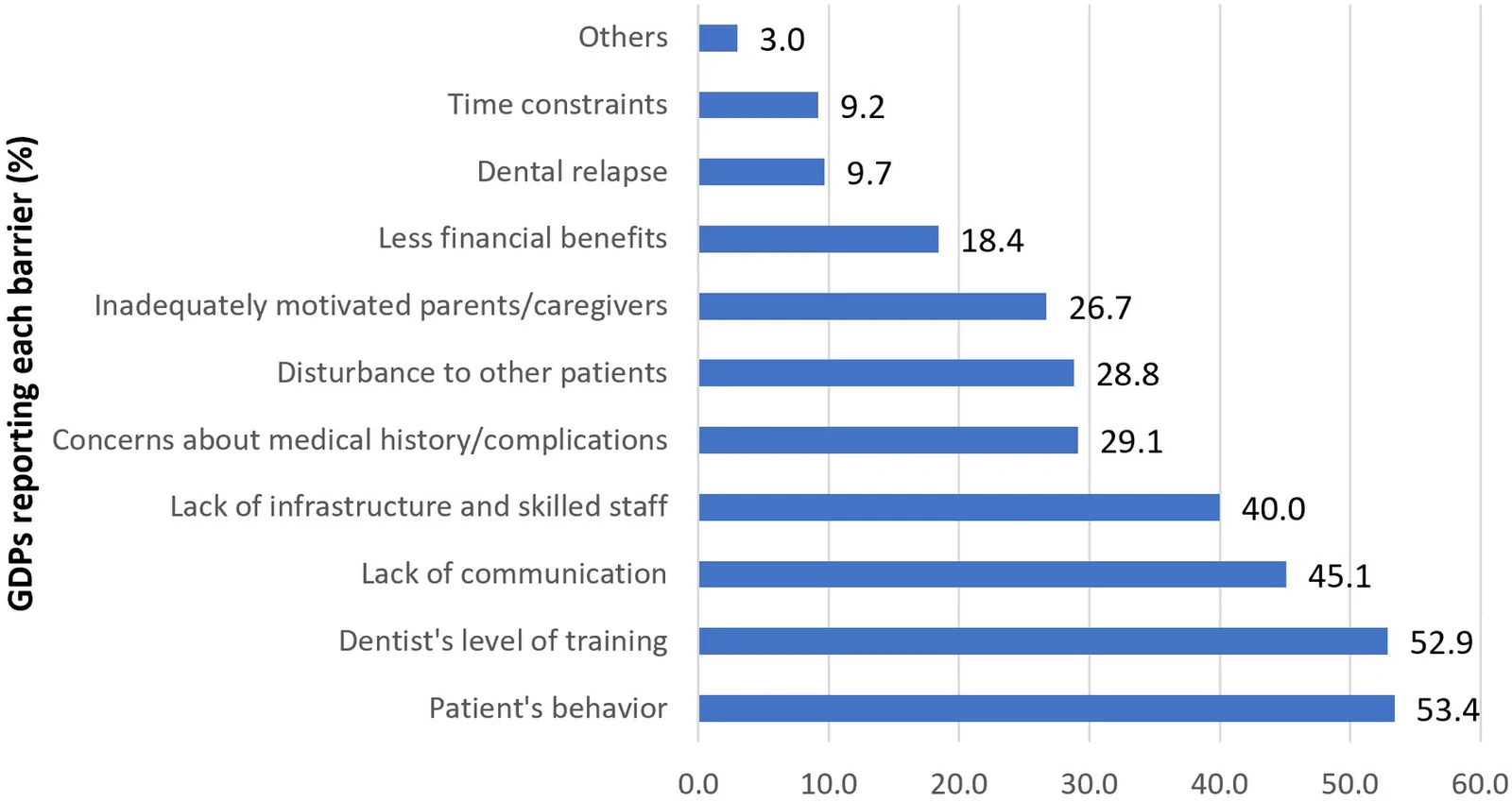
Perceived barriers to providing dental care to children with special healthcare needs (SHCN) in the UAE. GDP, general dental practitioner; SHCN, special healthcare needs.

The most frequently reported barriers were patient behavior (53.4%) and dentists' level of training (52.9%), followed by communication difficulties (45.1%) and infrastructure or lack of skilled staff (40.0%) (Figure 2).

GDPs reported being most comfortable treating children with physical disabilities (50.0%) (Table 3). The most performed procedures were routine examinations (68.1%), oral hygiene instruction and preventive care (56.8%), and emergency treatment (55.3%) (Table 4).

**Table 3:** Categories of children with special healthcare needs (SHCN) that GDPs reported being most comfortable treating.

Category of special healthcare needs (SHCN)	*n* (%)
Physical disabilities	103 (50.0)
Behavioral/emotional impairments	90 (43.7)
Developmental disabilities	60 (29.1)
Medically compromised	60 (29.1)
Sensory impairments	50 (24.3)

Data are presented as frequency (percentage). Categories are not mutually exclusive; respondents could select more than one category.

GDP, general dental practitioner; SHCN, special healthcare needs.

**Table 4:** Types of dental procedures GDPs reported being most comfortable providing for children with special healthcare needs (SHCN).

Procedure	*n* (%)
Routine examination	139 (68.1)
Oral hygiene instruction/preventive care	117 (56.8)
Emergency treatment	114 (55.3)
Restorative treatment	96 (46.6)
Advanced procedures	75 (36.4)

Data are presented as frequency (percentage). Procedures are not mutually exclusive; respondents could select more than one option.

GDP, general dental practitioner; SHCN, special healthcare needs.

### Subgroup analyses

Subgroup analyses were exploratory and should be interpreted as associations rather than independent predictors, because multivariable regression analyses were not conducted. Significant differences were observed across practitioner characteristics. Female GDPs were more likely to report dentists' level of training as a barrier (*p* = 0.027), whereas male GDPs reported higher confidence in managing medical emergencies (*p* < 0.001). Female GDPs were also more likely to report that their clinics had adequate equipment (*p* = 0.012).

Non-UAE graduates were more likely to report better undergraduate preparation (*p* < 0.001), higher confidence in treating children with SHCN (*p* = 0.028), greater confidence in managing medical emergencies (*p* = 0.008), and greater supporting staff readiness (*p* = 0.017). UAE graduates were more likely to report that their clinics were adequately equipped (*p* = 0.013).

GDPs aged ≥40 years were more likely to report higher confidence in treating children with SHCN (*p* = 0.009). Those aged <40 years were more likely to report communication difficulties (*p* = 0.009) and financial concerns (*p* = 0.031) as barriers. GDPs with >5 years of experience were more likely to report better undergraduate preparation (*p* = 0.017) and higher confidence in treating children with SHCN (*p* = 0.008) compared with those with ≤5 years of experience.

## Discussion

This study provides UAE-specific evidence on GDPs' perceived barriers to dental care for children with SHCN. The main findings were that GDPs most frequently identified patient behavior, insufficient practitioner training, communication difficulties, and infrastructure or staffing limitations as barriers. Despite strong interest in further training, respondents reported only moderate confidence in treating children with SHCN and managing related medical emergencies, highlighting a gap between willingness and preparedness. A notable finding was the discrepancy between reported physical accessibility and broader clinical readiness: most respondents said their clinics were physically accessible, yet more than half reported that suitable equipment was unavailable. This suggests that physical access alone may be insufficient to ensure readiness for SHCN care.

The conceptual frameworks informed interpretation by situating the findings within interacting individual, provider, and system-level determinants of access. Accordingly, the observed findings—moderate practitioner confidence, preparedness concerns, limited equipment readiness, and subgroup differences—should not be viewed as isolated outcomes but rather as interconnected determinants operating across individual, provider, and system levels of access (6, 7, 16).

The predominance of behavioral challenges and training-related barriers is consistent with international evidence. Previous studies have shown that dentists often perceive managing behavior and communication in children with SHCN as particularly challenging, especially in the absence of adequate training (10, 11). Similarly, systematic reviews have identified insufficient clinical experience, communication barriers, and limited exposure to special care dentistry (SCD) during undergraduate training as key contributors to reduced provider confidence and limited-service provision (12). These findings indicate that access challenges are not solely related to patient complexity but are strongly influenced by the preparedness of the dental workforce.

In the UAE context, this suggests that workforce preparedness, rather than policy commitment alone, may be a key modifiable factor influencing GDPs' readiness to provide SHCN care.

A key finding of the present study is that GDPs demonstrated high willingness to improve, as reflected by strong support for integrating SCD into undergraduate curricula and high interest in further training. However, this willingness contrasts with the reported moderate confidence levels, indicating a need for more structured educational preparation rather than simply greater awareness. This aligns with previous literature showing that inadequate undergraduate exposure to SHCN patients leads to reduced competence and confidence among general dentists (8, 9). Recent evidence among dental students further supports improved SCD curricula and training to develop the knowledge and skills required for care of patients with special needs (17). Structured training and mentoring approaches may further improve practitioners' confidence and ability to provide care (13, 18).

These findings should not be interpreted as implying that undergraduate programs must train all students to independently manage advanced or complex SHCN cases. Rather, they support the need for foundational undergraduate exposure to disability awareness, communication, prevention, behavior guidance, reasonable accommodations, emergency preparedness, and appropriate referral (3, 8, 9, 17, 18). Advanced management of complex cases remains more appropriately addressed through postgraduate pediatric dentistry, special care dentistry, hospital-based training, and continuing professional development (3, 13, 19). This distinction is important because GDPs require sufficient competence to provide preventive and routine care, recognize when adaptations are needed, and refer complex cases appropriately, while specialist pathways remain essential for advanced care (3).

Another important observation is the discrepancy between reported clinic accessibility and clinical readiness. Although most respondents reported that their clinics were physically accessible, many indicated inadequate equipment, suggesting that physical access alone may not ensure readiness to provide SHCN care. This finding should be interpreted in relation to practice setting, as equipment availability and service readiness may differ between primary healthcare centers, private clinics, and hospital-based dental services. Effective care for children with SHCN requires not only physical access but also appropriate equipment, trained personnel, adapted care pathways, and clear referral options (3, 5, 19, 20). Future studies should examine clinic readiness by facility type, availability of referral pathways, access to sedation or general anesthesia services, and support from trained auxiliary staff. Such stratification would help distinguish barriers that are primarily provider-related from those reflecting the structure and resources of the practice environment.

These UAE findings are broadly consistent with emerging evidence from other Gulf settings, although direct comparisons are limited by differences in study populations and perspectives. In Saudi Arabia, caregiver- and provider-focused studies have reported barriers including fear of the dentist, child uncooperativeness, travel distance, inadequate clinic facilities, shortages of specialist pediatric dentists, referral inefficiencies, financial constraints, physical barriers, and professional training needs (21, 22). Evidence from Qatar similarly highlights limited caregiver awareness of available oral healthcare facilities and preventive care, as well as perceived dentist reluctance to treat children with disabilities (23). In Kuwait, parents of children with disabilities reported difficulty obtaining appointments and child cooperation challenges and rated dental services less favorably than parents of children without disabilities (24). Oman-specific dental literature was not identified in the present search; however, broader disability service reports describe gaps in data systems, cross-sectoral coordination, awareness of available services, and concentration of specialized services in major cities (25). Taken together, regional evidence suggests that access to dental care for children with disabilities is shaped not only by provider confidence but also by service awareness, clinic readiness, referral pathways, caregiver support, and system-level coordination.

The UAE contribution is therefore its provider-focused perspective, which complements caregiver- and system-level evidence from neighboring Gulf countries and highlights clinic readiness and referral support as practical targets for future service improvement.

Subgroup analyses further reinforce the role of experience and training. Older and more experienced GDPs, as well as those trained outside the UAE, reported higher confidence levels, whereas younger and less experienced practitioners perceived more barriers. This pattern is consistent with previous studies demonstrating that clinical exposure and accumulated experience enhance self-efficacy in managing patients with special needs (8, 11). Recent qualitative evidence also indicates that extended undergraduate SCD experience can strengthen students' confidence, critical thinking, and clinical care skills (18). These findings suggest that early-career GDPs may require additional structured support, including supervised clinical exposure, case-based learning, and mentoring.

## Limitations

This study has several limitations. First, the cross-sectional design prevents causal inference and does not establish whether practitioner characteristics directly influence perceived barriers or confidence. Second, the final sample of 206 participants did not reach the originally calculated target of 240, which may have reduced statistical power and precision, especially for subgroup analyses. Third, the use of convenience sampling and online dissemination may have introduced selection bias. Dentists with greater interest in SHCN care or greater engagement with professional networks may have been more likely to participate, while less-engaged practitioners or those in underrepresented practice settings may have been less likely to respond. Fourth, because the survey was disseminated through open professional channels, the total number of dentists reached could not be determined and a conventional response rate could not be calculated. This limits assessment of nonresponse bias. Fifth, the study relied on self-reported perceptions and did not assess actual clinical behavior, objective competence, clinic audits, or patient outcomes. Responses may therefore be affected by recall bias, social desirability bias, or differences in respondents' interpretation of survey items. Sixth, although practice setting was collected, equipment availability and clinic readiness were not stratified by facility type; therefore, differences between primary healthcare centers, private clinics, and hospital-based dental services could not be examined. Seventh, although the questionnaire was adapted from a previously validated instrument and demonstrated acceptable internal consistency, formal construct validation in the UAE context was not performed. Finally, subgroup analyses were exploratory and unadjusted. Multivariable logistic or ordinal regression analyses were not conducted, and no adjustment for multiple comparisons was applied. Therefore, observed subgroup differences should be interpreted cautiously as associations rather than causal effects or independent predictors.

## Conclusions

UAE GDPs reported that barriers to providing dental care for children with SHCN are multifactorial and include behavioral challenges, perceived training gaps, communication difficulties, and limitations in clinic readiness. Although many respondents supported further education and reported physical clinic accessibility, confidence was generally moderate and suitable equipment was not consistently available. These findings highlight areas for improvement in foundational education, continuing professional development, clinic preparedness, and referral support, while recognizing that stronger causal and policy conclusions require further research using representative sampling, objective readiness assessments, and patient-centered outcomes.

## Recommendations

From an educational perspective, the findings support strengthening foundational undergraduate exposure to SHCN care, including disability awareness, communication, behavior guidance, preventive care, emergency preparedness, and referral decision-making. Undergraduate education should focus on core competencies and supervised exposure rather than independent management of advanced cases. More complex clinical management should remain within postgraduate specialist training, hospital-based care, and structured continuing professional development.

From a clinical perspective, the findings suggest the value of improving clinic readiness through appropriate equipment, trained support staff, longer or flexible appointments where needed, communication adaptations, and clear referral pathways. These recommendations should be interpreted as practical implications generated by a cross-sectional perception survey rather than evidence of causal effectiveness.

At the health-system level, the findings may inform stakeholder discussions on inclusive oral healthcare, awareness of existing legislation and services for People of Determination, and development of coordinated referral networks. However, policy and licensing recommendations should be considered cautiously and should be supported by further studies assessing patient outcomes, service capacity, and implementation feasibility.

## Data Availability

The raw data supporting the conclusions of this article will be made available by the authors, without undue reservation.
